# Medical Management of the Near-Narrowed Internal Auditory Canal Pathology in the Adult Population: A Preliminary Study

**DOI:** 10.3390/jcm14010253

**Published:** 2025-01-03

**Authors:** Pierre Reynard, Samar A. Idriss, Eugenia Mustea, Aïcha Ltaief-Boudrigua, Eugen Constant Ionescu, Hung Thai-Van

**Affiliations:** 1 Department of Audiology and Otoneurological Explorations, Civil Hospitals of Lyon, 69003 Lyon, France; samar.a.idriss@hotmail.com (S.A.I.); eugenia.mustea@chu-lyon.fr (E.M.); hung.thai-van@chu-lyon.fr (H.T.-V.); 2 Paris Hearing Institute, Institut Pasteur, Inserm U1120, 75015 Paris, France; 3 Department of Physiology, Claude Bernard Lyon 1 University, 69003 Lyon, France; 4 Department of Otolaryngology, Dar Al Shifa Hospital, Hawally 13034, Kuwait; 5 Department of Otolaryngology and Head and Neck Surgery, Holy Spirit University of Kaslik, Eye and Ear Hospital, Beirut 1201, Lebanon; 6 Department of Radiology, Hospices Civils de Lyon, 69003 Lyon, France; aicha.ltaief-boudrigua@chu-lyon.fr

**Keywords:** vestibular paroxysmia, neuro vascular cross compression, narrowed internal auditory canal, antiepileptic therapy

## Abstract

**Background/Objectives**: Objective: To discuss therapeutic outcomes in patients with symptomatic near-narrow internal auditory canal (NNIAC). **Methods**: We retrospectively analyzed the records of 26 symptomatic patients diagnosed with NNIAC, who had been treated with anti-epileptic drugs. In addition to clinical and radiological data, we recorded I–III latencies of auditory brainstem responses prior to and after medical therapy. **Results**: Among a total of 48 patients with NNIAC, 26 patients were included. Oxcarbazepine, Gabapentin, and Lamotrigine were prescribed among 19 (73%) patients, 6 (23%) patients, and 1 patient (4%), respectively. After treatment, 24 (92.3%) patients described improvement of vestibular symptoms, and 16 (76.2%) reported improvement of auditory symptoms. After treatment with antiepileptic drugs, ipsilateral IPL I-III latencies decreased (less than 2.3 ms) in 16 (84.2%) patients (23 ears out of 42). **Conclusions**: A low dose of anti-epileptic monotherapy for NNIAC could be effective over the long term and is generally well-tolerated. Further studies are needed to provide more solid evidence of the efficacy and safety of anti-epileptic drugs on a larger number of patients with NNIAC.

## 1. Introduction

Jannetta et al. hypothesized a relationship between “disabling positional vertigo” and the presence of a vascular anatomical compressing of the cochleo-vestibular nerve (CVN) at its root entry zone [1,2]. This condition was defined as Neurovascular Cross Compression (NVCC) of the CVN. Vestibular Paroxysmia (VP) corresponds to short attacks of spinning or non-spinning vertigo triggered by head movements or changes of position [3]. Barany Society committee diagnostic criteria for VP included at least ten spontaneous vertigo attacks with or without rotation, lasting less than 1 min, responding to antiepileptic treatment with carbamazepine (CBZ) or oxcarbazepine (OXC), with no further argument for another neurotologic diagnosis [4]. Further vestibular (including BPPV), neurological or vascular differential diagnoses were well detailed by the authors.

VP usually refers to the presence of a vascular structure in the cerebellopontine angle (CPA) in contact with the CVN causing a localized neuropathy [4,5]. The presence of a vascular structure partially compressing the CVN would lead to a local demyelination process and ephaptic discharges inducing brief vestibular and/or auditory symptoms [6]. However, this assumption is not supported by a recent 7T MRI study that showed no structural neural damage in patients with VP [7]. While microvascular decompression could improve VP symptoms in NVCC patients [1], medical management with low-dose antiepileptic drugs should be initially prescribed [3,8,9,10]. In view of its possible serious postoperative complications [4], microvascular decompression of the CVN is reserved for formal documented clinical and radiological cases with VP in patients who fail to respond to or tolerate antiepileptic drugs.

The link between NVCC and VP is still debated, given that healthy people can have radiological arguments for NVCC [8]. Chen et al. (2022) reported that 65% of radiologic NVCC did not meet the clinical criteria of VP, which was lower than expected. In their study, the response to antiepileptic drugs in VP patients with or without NVCC was indistinguishable. Also, it was suggested to initiate medical therapy in patients with clinical presentations consistent with VP, even if MRI results were negative [8]. In line with the present study, we reported a clinical entity similar to VP, in children and adults, in the absence of NVCC but in the presence of diminished internal auditory canals’ (IAC) diameters in axial and/or in coronal HRCT planes [11,12]. The diagnosis requires a careful radiological (HRCT and/or MRI) analysis of the IAC’s shape and diameters in the axial and coronal plane [12].

The narrowed IAC is a radiological (HRCT) term introduced by Valvassori, corresponding to a less than 2 mm diameter in the axial plane with hypoplastic or absent CVN [13,14,15]; therefore, we suggested the use of the “near” narrowed IAC (NNIAC), which is consistent with preserved morphometric characteristics of IAC on MRI [12]. Since axial cuts seemed to be less discriminating than coronal cuts, the NNIAC threshold values of 3.3 mm (in HRCT) and 2.9 mm (in MRI) were used in the present study [12]. As the near-narrowed IAC can generate VP-evocative symptoms, treatment with antiepileptic drugs appears to be effective not only in relieving dizziness but also in normalizing electrophysiological findings in some patients [11].

The aim of this study is to determine whether NNIAC, as a new etiology of VP in adults, can be treated similarly to NVCC. To achieve the latter purpose, data on vestibular and auditory symptoms, electrophysiological auditory brainstem responses, and radiologic measurements were collected and analyzed prior to and after initiation of treatment with antiepileptic drugs.

## 2. Materials and Methods

### 2.1. Population

Over a three-year period (January 2021 to January 2024), all patients treated for NNIAC syndrome [12] in our department who had benefitted from electrophysiological auditory exploration using ABR, and radiological exploration using MRI and CT were retrospectively recruited. Patients with severe impairment due to dizziness or non-pulsatile tinnitus were proposed antiepileptic drugs on the basis of Dizziness Handicap Inventory (DHI > 54) [16] and the Tinnitus Handicap Inventory (THI > 58) [17].

Exclusion criteria were: patients younger than 18 years old, adults with a personal or a family history of headache or migraine, motion sickness, severe hearing loss; patients with previous consumption of ototoxic drugs, middle or inner ear abnormalities, central disorders, ophthalmic or vergence anomalies, psychological disorders, or systemic pathologies. Patients with an image of a clearly pathogenic vascular bundle diverting the CVN from its course or other vascular malformation were also excluded. Patients with hyperacusis were not included as well (non-obtainable ABR).

The investigation adhered to the principles of the Declaration of Helsinki (https://www.wma.net/wp-content/uploads/2016/11/DoH-Oct2013-JAMA.pdf (accessed on 23 December 2024)). Written informed consent was obtained from the patients.

### 2.2. Audio-Vestibular Assessment

After collecting their detailed medical data and performing a thorough neurotological examination, all patients were subject to a comprehensive audio-vestibular assessment. Otoscopy and tympanometry were verified in each subject. Each participant had a pure tone audiometry (PTA; Callisto, Interacoustics, Middelfart, Denmark). PTA thresholds were recorded while each patient was seated in a soundproof booth (ISO 8253, https://www.iso.org/standard/43601.html (accessed on 1 January 2025) [18]). ABR were recorded in line with NVCC Moller’s criteria [19,20]. In certain cases of NVCC accompanied by auditory symptoms, I-III interpeak latency (IPL) has been found to correlate positively with the ipsilateral hearing loss on the tinnitus frequency, and this tends to worsen with time [20]; the I-III IPL was considered prolonged when it exceeded 2.3 ms.

As part of vestibular evaluation, each patient performed a videonystagmoscopy to document the presence of spontaneous nystagmus and/or positional nystagmus, and hyperventilation-induced nystagmus (HVIN) recording (see [6,21]). A full neuro-otological work-up, including fistula test and Valsalva maneuver was performed. BPPV diagnostic maneuvers were verified (negative) for all included patients. The vestibular assessment in all groups included a videonystagmography (Ulmer^®^, Synapsis SA, Marseille, France) with bilateral caloric water stimulation. A Video Head Impulse Test (VHIT, GN Otometrics^®^, Taastrup, Denmark) was also performed for each semicircular canal. Saccular otolithic function was assessed on cVEMPs (NavPro, Bio-Logic^®^, Mundelein, IL, USA) elicited in air conduction (AC, at 750 Hz tone bursts stimulation) during sternocleidomastoid contraction. An absent response was defined by a non-reproducible p13–n23 over two attempts.

### 2.3. Radiological Assessment

**Figure 1:**
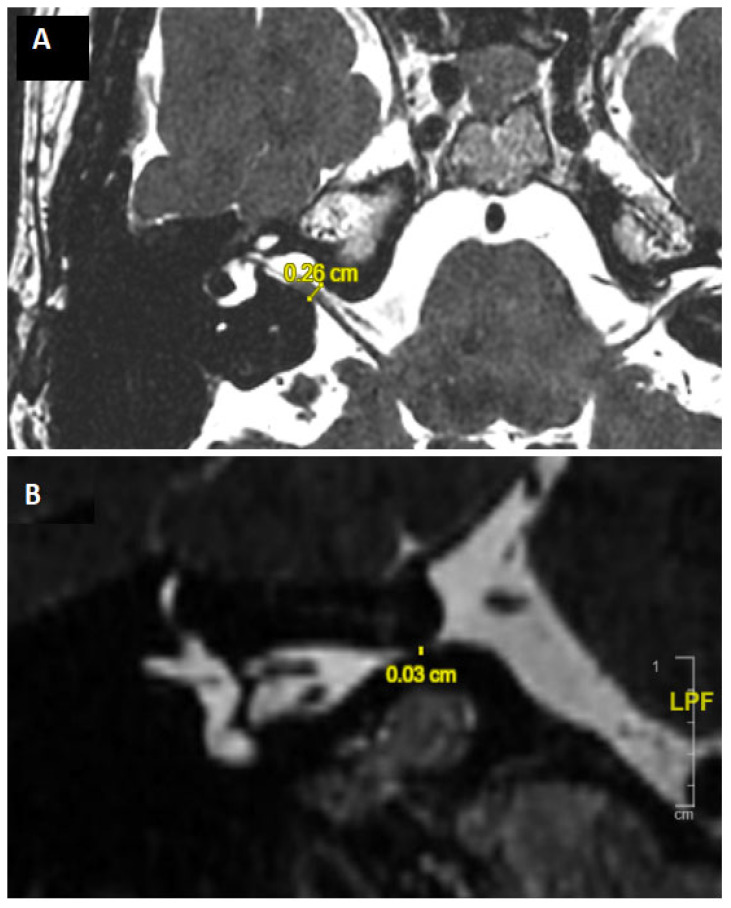
3D T2 high-resolution MRI (DRIVen Equilibrium pulse) of the IAC; measurement of IAC diameter in axial (**A**) and coronal plane (**B**) evoking a right severely narrowed IAC. IAC: internal auditory canal. Echo time 157, Repetition time 1000, slices thickness 0.4, Turbo factor 40, Matrice 500 × 500, voxel size: 0.4 × 0.4 isotropic).

**Figure 2:**
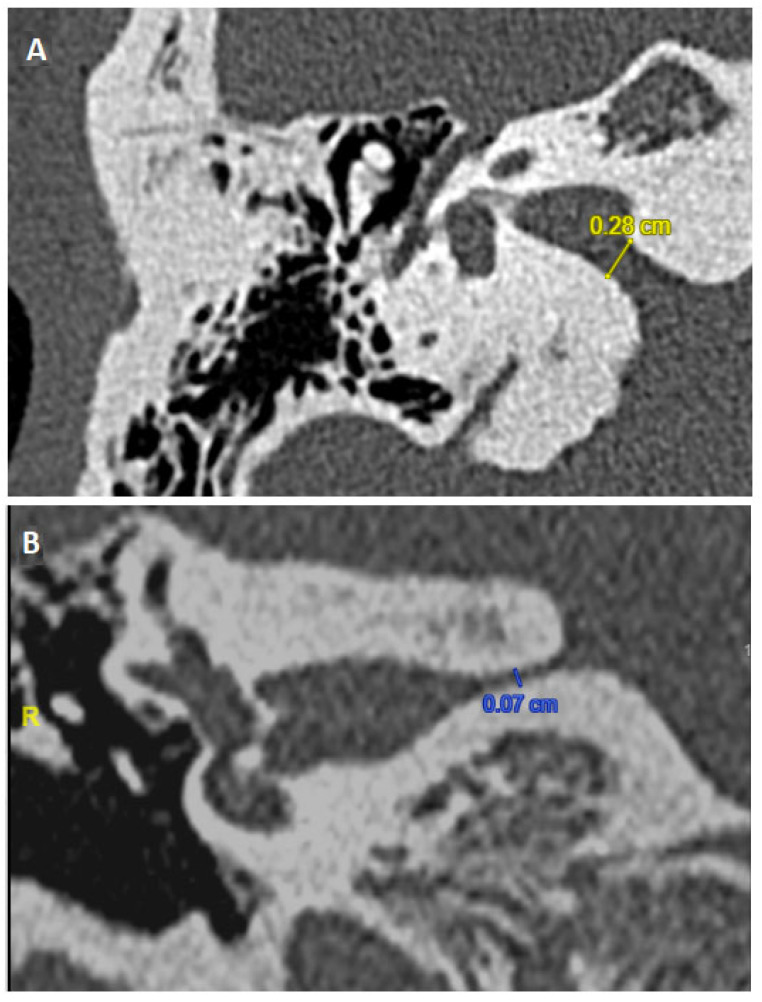
Tomodensitometry (ultrahigh resolution at 140 kV and 200 mAs/section) of temporal bone (same patient, not included). Slices were acquired helically in the axial plane at a nominal 0.625 mm slice thickness with a 50% overlap of 0.312 mm. The primary images were retargeted to the axial and coronal planes of the lateral semicircular canal to a 60 mm field of view with a 512 matrix for an isometric voxel. Narrowing is confirmed in axial (**A**) and coronal plane (**B**).

All patients underwent a cerebral and IAC 1.5 and/or 3 Tesla MRI to eliminate central pathologies and check for sufficient CVN development. The CVN course in the CPA and IAC was also closely analyzed to identify and exclude patients with images suggestive of NVCC (Figure 1). When narrowing of the IAC was suspected, patients underwent HRCT of the temporal bones (Figure 2). In line with our previous studies, a “near” narrowed IAC was suspected if the anteroposterior or cranio-caudal IAC diameter was measured less than 3 mm [11] (see [12] for radiological algorithm description). All radiological analyses and measurements were carried out by the same radiologist (ALB) and double-verified by a senior neurotologist (EI).

### 2.4. Therapeutic Strategies

Antiepileptic therapy was initiated when symptoms were troublesome, recurrent, and disabling according to DHI and THI scores. As indicated by Barany Society, a therapeutic trial similar to that proposed for VPs with NVCC, a low-dose CBZ (200–800 mg/day) or OXC (300–900 mg/day) was considered as a first line therapy [4]. In case first-line therapy was ineffective or non-tolerated, Gabapentin was opted for as suggested by some authors [10,22,23]. The initial duration of treatment was set at 6 weeks. Electrophysiological (ABR) assessments were performed to document the efficacy of treatment and to validate the diagnosis of VP. Five auditory and vestibular symptoms (vertigo, balance disorders, tinnitus, fullness of the ear, hypoacusis) were recorded before and after treatment, and patients were simply asked to comment on whether their symptoms had improved, remained stable or deteriorated. Biological monitoring (liver and renal function lab tests) was carried out every 15 days throughout the treatment.

## 3. Results

Overall, 48 patients with a narrowed IAC were selected. A total of 26 patients were included. Patients with confounding variables, incomplete medical records, or loss of follow-up were not considered (22 patients).

### 3.1. Demographic Data

The demographic and auditory characteristics of the population are shown in Table 1. Among 26 patients, 7 (27%) were males and 19 (73%) were females, with a mean age of 43.8. The majority of patients have auditory responses with normal ranges by age (96%), i.e., air conduction PTA thresholds less than 15 dB HL at octave intervals from 0.25 to 8 kHz. Ten (38%) patients presented spontaneous horizontal nystagmus, and 16 (61.5%) patients had no spontaneous nystagmus. It’s worth noting that of those with spontaneous nystagmus (n = 10), the latter was accentuated in 9 (90%) patients and reduced in 1 (10%) patient after the hyperventilation maneuver. Of those free of spontaneous nystagmus (n = 16), the hyperventilation maneuver was positive in 11 (68.7%) patients (9 horizontal, 1 vertical).

### 3.2. Diagnostic (Radiologic) Data

Diagnostic assessment, including radiologic findings, was obtained (Table 2). All patients met the clinic criteria of probable VP. The IAC measurements were analyzed on the MRI showing a narrowed canal on coronal planes in 15 (57.6%) patients or on both axial and coronal planes in 11 (42.3%) patients. The measurements were interpreted more precisely on the HRCT, which showed narrowing on either axial (7.7%), coronal (84.6%), or both the axial and coronal (7.7%) planes. While 16 (61.5%) patients presented bilateral narrowing of the IAC (32 ears), 10 patients (38.5%) presented unilateral narrowing (10 ears).

### 3.3. Therapeutic Data

Following the diagnostic assessment, a treatment with antiepileptic drugs was prescribed to alleviate symptoms of incapacitating attacks (Table 3). Therefore, Oxcarbazepine (OXC), Gabapentin, and Lamotrigine were prescribed to 19 (73%) patients, 6 (23%) patients, and 1 patient (4%), respectively. Before treatment, vestibular symptoms (vertigo, balance disorders) were present in all (100%) patients, and auditory symptoms, including non-pulsatile intermittent tinnitus, hypoacusis, and/or ear fullness, were present in 21 (80.8%) patients. After treatment, 24 (92.3%) patients described improvement of vestibular symptoms, and 16 (76.2%) an improvement of auditory symptoms. While 3 (11.5%) patients complained of reappearance of symptoms directly after withdrawal of treatment, the remaining patients were asymptomatic for a total duration of 12 weeks. Treatment-related adverse effects were rare. Few patients (6 patients) reported intolerance to the first-line (OXC) therapy (i.e., nausea, insomnia, blurred vision, and/or headache) and switched to an alternative therapy (Gabapentin). Due to lack of improvement on OXC, 1 patient was treated with lamotrigine by a neurologist (outside our unit), in the hypothesis of epilepsy. Because of its efficacy on vestibular symptoms, this treatment was continued even though the diagnosis of epilepsy had not been confirmed.

In addition to the post-therapeutic clinical assessment, objective assessment was based on normalization of ipsilateral and contralateral IPL I–III latencies (see Table 3). Before treatment, among patients with bilateral radiologic narrowing of IAC, 4 (25%) patients did not show bilateral prolonged IPL I–III latencies; 5 (31.25%) patients had only unilateral prolonged IPL I-III latencies, of which 4 had also contralateral prolonged IPL I-III latencies. Among those with unilateral narrowing of IAC (10 ears), 7 (70%) patients had ipsilateral prolonged IPL I-III latencies, of which 5 patients had contralateral prolonged IPL I-III latencies. After treatment with antiepileptic drugs, ipsilateral IPL I-III latencies decreased (less than 2.3 ms) in 16 (84.2%) patients (23 ears out of 42). Overall, among all patients with normal or pathological I-III latencies, the latter were reduced by >0.05 ms in 30 (57.7%) out of 52 ears and were increased by >0.05 ms in 3 (5.7%) out of 52 ears.

## 4. Discussion

We recently introduced the NNIAC as a distinct clinical entity, corresponding to VP-like cochleovestibular symptoms in patients with unilateral or bilateral narrowed IACs and a normal size CVN on MRI. In the present study, we presented clinical and electrophysiological evidence suggesting that patients suffering the above-mentioned condition may improve with antiepileptic treatment (at a dosage identical to that suggested for VP in NVCC).

### 4.1. Clinical Considerations

Of a total number of 26 patients, 19 were female. The literature on VPs does not find a gender predisposition [4], but it is based on VP patients for whom an image of NVCC is usually considered; moreover, our sample is too small to draw any conclusions. All NNIAC patients showed clinical improvement of auditory (76.2%) and vestibular (92.3%) symptoms. It is essential here to keep in mind that NNIAC patients may have difficulty reconciling their symptoms, making detailed analysis of clinical improvement difficult without the risk of reporting bias. However, Chen et al. (2022) argued that control of auditory symptoms in VP may require higher doses of OXC than for vestibular symptoms [8].

It should be emphasized that we were unable to measure this improvement using validated questionnaires, notably the DHI [16] and the THI [17]. These questionnaires were simply used for inclusion. In fact, since VP is characterized by brief episodes of vertigo, these types of questionnaires tend to underestimate the patients’ discomfort. A very simple evaluation of the 5 reported symptoms (vertigo, balance disorder, tinnitus, ear fullness, hypoacusis) was used, with the patient simply having to report improvement, deterioration or stability of symptoms. This evaluation gives an idea of the clinical improvement brought to the patient, but is not sufficient to confirm it. It was clear that patients with VP can experience a significant deterioration of their quality of life, leading them to claim an effective medical treatment, even if the anathema “antiepileptic drug” may initially destabilize some of them. Therefore, larger studies with adapted and validated subjective assessment tools and objective outcomes are needed to better measure the real impact after treatment.

Compression of the rostroventral VIII nerve (vestibular nerve) is in correlation with vertigo, whereas the compression of the caudal surface of the VIII nerve (cochlear nerve) appears to be associated with a complaint of tinnitus. In patients complaining of both vestibular and acoustic symptoms, compression of both vestibular and cochlear nerves has been reported [23,24]. Although vestibular symptoms (92.3%) appeared to be more encountered than auditory symptoms (76.2%) in the present study, the risk of symptoms report bias has already been highlighted. It is worth noting that the auditory nerve encloses a greater number of fibers compared to the vestibular nerve, matching with the need for higher doses of anti-epileptic drugs to control auditory symptoms [25,26,27]. The latter is therefore more fragile and sensitive to extrinsic compression, however slight, by the bony structure (NNIAC) or a vascular structure (NVCC). In addition, at least distally within the IAC, the auditory fiber pathway rotates around the axis of the cochlear nerve [25,27]. These morphological and anatomical peculiarities of the CVN can reasonably explain the higher prevalence of vestibular symptoms in relation to auditory ones in this pathology. Further, as previously reported [12], unlike the neuropathy secondary to ephaps in NVCC (generated by intermittent compression of a soft, vascular structure by pulsations), neuropathy due to compression of a hard surface such as bone appears to be clinically different, with less paroxysmal, more continuous symptoms, such as the spontaneous nystagmus found on examination (which may be inhibited by hyperventilation). Moreover, the patients included in the previous article defining the NNIAC entity presented symptoms suggestive of probable VP according to the Barany criteria, i.e., with seizures lasting longer than definite VP (“duration less than 5 min”). These criteria are the same as those used to include patients in the present study.

### 4.2. Electrophysiological Considerations

Some researchers have proposed electrophysiological criteria for selecting patients with NVCC and “severe” tinnitus (no further description) for surgical decompression [19,28]. De Ridder et al. (2011) proposed ABR criteria (ipsilateral prolongation of interval I-III, contralateral prolongation of interval III-V, and decrease in amplitude of wave II peak) as elements of pathophysiological mechanisms explaining tinnitus [20]. Prolonged latencies of IPL I-III and wave III in VP patients strongly suggest pathological neurovascular contact of the CVN [4], which may provide arguments in favor of microvascular decompression surgery. To date, there are no randomized controlled trials for VP medical treatment, but response to CBZ or OXC supports the diagnosis [4,23,29]. In a retrospective study of 17 patients treated with carbamazepine or OXC for typewriter tinnitus associated with NVCC, Sun et al. (2023) studied the value of using IPL I-III prolongation to predict the risk of recurrence after treatment discontinuation. It was noticed that 7 patients had a loss of treatment-related benefit for typewriter tinnitus after discontinuation, and patients with I-III IPL greater than 2.4 ms were more likely to have a recurrence [30]. De Ridder D. et al. reported the absence of ABR changes during the first 2 years of tinnitus (not specified) associated with a NVCC. Following that period, a decrease of wave II amplitude and a prolongation of I-III IPL were documented [20]. These findings suggest electrophysiological modifications are not necessarily a constant criterion in symptomatic VP. In the present study, low doses of antiepileptic drugs were successfully prescribed in patients with bothersome symptoms (according to DHI and THI scores), resulting in relieving patients’ symptoms. Although the long-term administration of anticonvulsant drugs in epileptic patients was suspected to provoke delayed auditory conduction [31,32], we found a normalization of IPL I–III in 16 out of 19 patients. These results are in line with our previous paper suggesting that low doses of oxcarbazepine used in VP could lead to an improvement in symptomatology and electrophysiological parameters in NNIAC as a local neuropathy [11]. Despite the fact that some patients have an improved ABR pattern after therapeutic management with anti-epileptics, improvement was not systematic. All in all, it seems more prudent to still consider I-III IPL prolongation as an additional diagnostic or therapeutic efficacy criterion. Further, the decrease in amplitude of wave II peak is sometimes difficult to measure in relation to baseline. Some patients in the study had a flattened peak II before treatment, but no clear improvement could be demonstrated.

### 4.3. Radiological Considerations for NNIAC Diagnostic

To confirm the diagnosis of NNIAC, measuring the smallest diameter of the IAC in both the axial and coronal planes is recommended, preferably by HRCT or MRI of the temporal bone [12]. These measurements are best carried out using temporal bone HRCT with the following method: (1) evaluation of the smallest anteroposterior and craniocaudal IAC diameters, both in the axial and coronal planes, after measuring the IAC length, (2) description of bony abnormalities of the IAC walls, (3) evaluation of any significant angulation or deformation of the IAC in the craniocaudal and anteroposterior planes.

In addition, 3T high-resolution T2 MRI analysis should mention: (1) the presence or not of any modification in the perineural cerebrospinal fluid environment between the CVN and the bony canal, (2) any angulation between the cisternal pathway and intramedullary path of the CVN, (3) a significant angulation of the CVN in the IAC or at its meatus, (4) and evaluating the presence of a potential vascular structure compressing the CVN [12]. Furthermore, performing an analysis of fusion images between high-resolution T2 and HRCT of the temporal bones for a more accurate evaluation of anteroposterior and craniocaudal diameters of the IAC can be very useful, although it is not mandatory. This analysis can be helpful to rule out the presence of a vascular structure compressing the CVN within a narrowed IAC. Similarly, to the present study, it is important to focus on the coronal measurement of the IAC at its narrowest point, where a vascular-nerve and/or nerve-to-bone compression is most likely to occur. Indeed, the compression syndrome in NNIAC may correspond to a conflict between two anatomical elements: the content (normal-sized CVN) and the container (smaller, and therefore smaller-volume IAC) [12]. It is therefore logical to imagine that when a vascular loop in a narrowed internal auditory canal coexist, the probability of having specific symptoms is higher, and it is crucial to have an exhaustive reading of MRI images.

### 4.4. Concluding Remarks and Considerations for Further Research

Some limitations of this study should be noticed. First, this is a retrospective descriptive study; the duration of follow-up differed among patients. Additionally, dizziness frequency was self-reported and subject to recall bias. Scales were not used for assessing the severity of dizziness and tinnitus after treatment, such as the DHI and the THI. Further studies, with larger populations, are essential to provide more solid evidence on the efficacy and safety of anti-epileptic drugs with this recently described pathology.

In line with the literature, including recommendations from the Barany Society [4], we proposed a therapeutic trial similar to that proposed for VP patients with NVCC, i.e., low-dose CBZ or OXC. There are still no high-level evidence studies on the treatment of VPs with NVCC by antiepileptic drugs, in a sufficient sample. Only a few studies have investigated the improvement of antiepileptic drugs in patients with NVCC [6,8,33,34,35,36,37]. The molecules used were OXC alone [8,35], CBZ alone [37] or a comparison of the 2 [6,33,34,36]. The samples of patients treated were fairly limited. In addition, the doses used were highly variable. These studies reported an improvement in symptoms, rarely significant [33,34,35]; no study has shown OXC to be more effective than CBZ, but it may be better tolerated: OXC was developed from a structural variation of CBZ with the aim of limiting side effects (blocking effect on sodium channels at much lower concentrations in vitro). Unlike CBZ, OXC metabolism is only weakly induced or inhibited by the cytochrome P-450 system [38]. This may explain why there are fewer interactions with other drugs, which is why we preferred this molecule. In case of intolerance to OXC or CBZ, other sodium channel blockers such as phenytoin, valproic acid, and gabapentin were also used; however, there is still no study data available [4,8,36].

Although prescribed by a small number of practitioners in France, based on recently published data, the prescription of antiepileptic drugs for VP has not been validated yet. An important practical limitation is to provide the French ENT practitioners the ability and legality to prescribe the aforementioned neuroprotective drugs (even though it has become possible in certain ENT departments whose labelling includes the word “neurotology”), which is currently reserved for neurologists, among whom NNIAC is not a well-known pathology, even in referral university centers.

## 5. Conclusions

The need to disseminate knowledge about NNIAC to caregivers of various specialities, including ENT specialists, radiologists, and neurologists, is fundamental in diagnosing and treating patients with VP. This will have a significant medical, social, and economic impact, not only by reducing diagnostic timelines but also by reducing multi-consultations, patients’ anxiety, and self-treatment.

## Figures and Tables

**Table 1:** Demographic data and clinical elements. HVIN: hyperventilation-induced nystagmus, HFSNHL: High-frequency sensorineural hearing loss. Direction of spontaneous nystagmus is described with reference to the fast phase.

	Sex	Age	Pure Tone Audiometry		Spontaneous Nystagmus (Direction)	HVIN
			Right	Left		
1	M	48	Mild SNHL	Mild SNHL	Left	+ (accentuation)
2	F	47	Normal	SNHL	Left	+ (accentuation)
3	F	37	Normal	Normal	Right	+ (accentuation)
4	F	45	Normal	Mild HF SNHL	0	+ (apparition)
5	F	46	Normal	Mild HF SNHL	Left	+ (accentuation)
6	M	44	Normal	Mild SNHL	Left	+ (diminution)
7	M	72	Presbycusis	Presbycusis	0	-
8	F	78	Mild SNHL	Normal	Left	+ (accentuation)
9	F	18	Normal	Normal	0	-
10	M	65	Presbycusis	Presbycusis	Left	+ (accentuation)
11	F	45	Normal	Normal	Left	+ (accentuation)
12	M	48	Normal	Normal	0	-
13	F	41	Normal	Normal	Left	+ (accentuation)
14	F	54	Normal	Normal	0	-
15	F	21	Mild SNL	Normal	0	+ (apparition)
16	F	21	Mild SNHL	Mild SNHL	0	+ (apparition)
17	F	37	Normal	Normal	0	+ (apparition)
18	F	49	Normal	Normal	0	-
19	F	49	Normal	Normal	0	+ (apparition)
20	F	70	Severe SNHL	Normal	0	+ (apparition)
21	F	23	Normal	Normal	0	+ (apparition)
22	M	29	Normal	Normal	Right	+ (accentuation)
23	F	41	Presbycusis	Mild HF SNHL	0	+ (apparition)
24	F	46	Normal	Normal	0	+ (apparition)
25	M	38	Normal	Normal	0	+ (apparition)
26	F	28	Normal	Normal	0	-

**Table 2:** Radiologic data of the patients in both the axial and coronal planes. MRI: magnetic resonance imaging; HRCT: high-resolution computed tomography; IAC: internal auditory canal.

	MRI Measurement—Right IAC Diameter (mm)		MRI Measurement—Left IAC Diameter (mm)		HRCT of Temporal Bones Narrowing Laterality	Right IAC Diameter (mm)		Left IAC Diameter (mm)	
	Axial	Coronal	Axial	Coronal		Axial	Coronal	Axial	Coronal
1	2.4	6	5	2.1	Bilateral	5.2	1.9	4.6	2.2
2	9	1.9	6	1.8	Bilateral (L > R)	3.4	2.1	1	2.1
3	6	2	13.1	2.2	Bilateral	3	2.3	5	2.4
4	0	2.4	2	1.7	Bilateral (L > R)	3.3	2.9	5	2.3
5	4.6	3.8	7	2.8	Left	3.3	2.9	5	2.3
6	9	4.2	7	2.8	Left	7	4.3	2.9	9
7	9	1.6	2	2	Bilateral (L > R)	3.4	2.7	2	2.9
8	9	2.3	8	2.1	Bilateral (R > L)	4.1	2.5	5	2.8
9	5	2.1	7	2.5	Bilateral	3.8	2.2	1	2.8
10	1	2.1	3	3	Left	4	3	0	3.2
11	0	2.4	2	1.5	Bilateral (L > R)	3.3	2.9	5	2.1
12	1	2.9	7	2.8	Right	3.8	2.7	9	3.4
13	1	2.6	8	2.1	Bilateral (L > R)	3.9	2.7	2	2.3
14	1	1.4	1	1.1	Bilateral (L > R)	2.8	1.8	3	1.3
15	6	2.8	7	1.8	Left	4.8	3.1	5	2.2
16	2.8	1.8	2	2.6	Bilateral (R > L)	3.9	2	5	2.8
17	5	2.1	9	2.2	Bilateral	4.2	2.9	8	2.4
18	2	2.3	1	2.1	Left	4	3	3	2.6
19	6	2.1	2	1.9	Bilateral (R > L)	3.6	2.2	3	2.1
20	5	2.5	6	2.4	Right	4.1	2.5	7	3.1
21	13.2	2.1	7	2.9	Right	3.3	2.3	9	3.1
22	7	1.8	7	2	Bilateral	3.7	2.6	4	2.6
23	6	2.5	8	2.1	Right	4.8	2.7	8	3.2
24	8	2.2	7	2.3	Bilateral	3.4	2.3	9	2.5
25	3.5	2.6	4.1	2.8	Right	3.9	2.9	5.5	3.3
26	4	2.3	4	2	Bilateral	4	2	4	1.8

**Table 3:** Therapeutic data for each patient, before and after treatment. ABR: auditory brainstem responses. V: vertigo; BD: balance disorder; T: tinnitus (non pulsatile, intermittent). EF: ear fullness; H: hypoacusis.

	Pre-Treatment ABR (I–III Interval, ms)		Clinical Signs (Vestibular/Auditory)	Treatment (Dose in mg, per Day)	Post-Treatment ABR (I–III Interval, ms)		Clinical Evolution
	Right	Left			Right	Left	
1	2.42	2.50	V, BD/T, H	Oxcarbazepine 300	2.40	2.12	Improvement
2	2.48	2.48	V, T/H	Oxcarbazepine 450	2.42	2.20	Improvement (V)
3	2.28	2.40	V, BD/EF, T	Gabapentin 900	2.25	1.92	Improvement (V, BD)
4	1.72	2.24	V, BD/T	Gabapentin 900	2.12	1.76	Improvement
5	2.44	2.48	V/T	Gabapentin 900	2.25	2.17	Improvement (T)
6	2.36	2.36	V/T, H	Oxcarbazepine 450	2.10	2.25	Improvement
7	2.75	2.33	V/T	Oxcarbazepine 450	2.33	2.08	Improvement
8	2.17	2.12	V/T, H	Oxcarbazepine 600	2.08	2.12	Improvement
9	2.37	2.37	V/0	Oxcarbazepine 300	1.87	2.07	Improvement
10	2.00	2.12	V/0	Oxcarbazepine 150	2.00	2.00	Improvement
11	2.12	2.24	BD/T	Gabapentin 900	2.12	1.76	Improvement
12	2.44	2.20	V, BD/T	Oxcarbazepine 150	2.31	2.20	Improvement
13	2.17	2.17	V, BD/T	Oxcarbazepine 150	2.17	2.12	Improvement
14	2.25	2.31	BD/T	Oxcarbazepine 300	2.25	2.04	Improvement (T)
15	2.54	2.46	V/T	Oxcarbazepine 300	2.62	2.46	Improvement
16	2.42	2.42	V/T	Oxcarbazepine 300	2.36	2.44	Improvement
17	2.42	2.29	V, BD/T	Oxcarbazepine 600	1.96	2.00	Improvement
18	2.33	2.31	V, BD/0	Gabapentin 900	2.31	2.32	Improvement
19	2.44	2.24	V, BD/T	Oxcarbazepine 300	2.17	2.17	Improvement
20	2.66	2.00	BD/T, EF	Gabapentin 900	1.83	2.00	Improvement (BD)
21	1.96	1.96	V, BD/0	Oxcarbazepine 300	1.95	2.00	Improvement
22	2.36	3.00	BD/T	Oxcarbazepine 450	2.40	2.54	Improvement (BD)
23	2.04	2.04	V, BD/T	Oxcarbazepine 150	2.17	2.08	Improvement (BD)
24	2.04	1.96	V, BD/T	Oxcarbazepine 450	2.04	2.04	Improvement (V, BD)
25	2.42	2.62	V, BD/T	Lamotrigine 150	2.29	2.58	Improvement (V)
26	2.42	2.34	V, BD/0	Oxcarbazepine 450	2.29	2.17	Improvement

## Data Availability

The data presented in this study are available on request from the corresponding author. The data are not publicly available due to ethical, legal, and privacy issues.
